# Tritesting in Battor, Ghana: an integrated cervical precancer screening strategy to mitigate the challenges of multiple screening visits and loss to follow-up

**DOI:** 10.3332/ecancer.2023.1645

**Published:** 2023-12-08

**Authors:** Kofi Effah, Ethel Tekpor, Comfort Mawusi Wormenor, Nana Owusu Mensah Essel, Seyram Kemawor, Edna Sesenu, Stephen Danyo, Yohane Teye Kitcher, Gifty Belinda Klutsey, Georgina Tay, Faustina Tibu, Kofi Antwi Abankroh, Bernard Hayford Atuguba, Patrick Kafui Akakpo

**Affiliations:** 1 Catholic Hospital, PO Box 2, via Sogakope, Battor, Volta Region, Ghana; 2 Department of Emergency Medicine, Faculty of Medicine and Dentistry, College of Health Sciences, University of Alberta, 730 University Terrace, Edmonton T6G 2T4, Canada; 3 Department of Pathology, Clinical Teaching Center, School of Medical Sciences, University of Cape Coast, PMB, Cape Coast, Ghana; a https://orcid.org/0000-0003-1216-2296; b https://orcid.org/0000-0001-5494-5411; c https://orcid.org/0000-0003-0356-0663

**Keywords:** human papillomavirus infection, uterine cervical neoplasm, cytology, Papanicolaou test, colposcopy, human papillomavirus DNA test

## Abstract

**Background:**

Human papillomavirus (HPV) DNA testing is more sensitive than cytology for detecting cervical precancer; however, increasing reports of high-risk HPV (hr-HPV)-negative cases of cervical intraepithelial neoplasia (CIN) and even malignancy motivate the use of combined testing. We present our experience with ‘tritesting’, defined as the performance of HPV DNA testing, cytology and visual inspection in a single session at the Cervical Cancer Prevention and Training Centre, Ghana. We further determined the prevalence rates of hr-HPV infection, abnormal cytology and cervical lesions among women screened using tritesting.

**Methods:**

This descriptive retrospective cross-sectional study assessed all women screened via tritesting between April 2019 to April 2023. HPV DNA testing was performed using the Sansure MA-6000, GeneXpert or AmpFire platforms. Visual inspection was performed using enhanced visual assessment mobile colposcopy or visual inspection with acetic acid. Liquid-based cytology was performed using cervical samples taken with a Cervex-Brush® and fixed in PreservCyt, while samples for conventional cytology were taken using an Ayre spatula and cytobrush.

**Results:**

Among 236 women screened (mean age, 39.1 years (standard deviation, 10.9)), the overall prevalence rates of hr-HPV infection and cervical lesions were 17.8% (95% confidence interval (CI), 13.1–23.3) and 11.9% (95% CI, 8.0–16.7), respectively. Cytology yielded findings of atypical squamous cells of undetermined significance or worse in 2.5% (95% CI, 0.9–5.5) of women. Histopathology following loop electrosurgical excision procedure revealed CIN I (tritest positive) and CIN III (hr-HPV-positive, visual inspection ‘positive’, cytology-negative) in one woman each. Factors independently associated with hr-HPV infection among ‘tritested’ women were age ≥ 39 years, tertiary level of education and current contraceptive use. Twenty-seven out of 39 hr-HPV-positive women (69.2%; 95% CI, 52.4–83.0) showed a type 3 transformation zone and would have needed to be recalled for a cytologic sample to be taken in a ‘see and triage’ approach with HPV DNA testing and a visual inspection method.

**Conclusion:**

This study brings tritesting into the spotlight, as an alternative to other methods, particularly for women who prefer this due to the advantage of a single visit to a health facility and being more cost-effective, if they have to travel long distances to access cervical screening services.

## Introduction

Cervical cancer is the second most common cancer affecting women in Ghana [1, 2]. However, as is the case in many low (middle)-income countries (LMICs), cervical precancer screening uptake in Ghana is low, with access to such services remaining largely opportunistic. Several developed countries have successfully implemented cytology-based cervical precancer screening programmes [3]. This approach, however, has been difficult to implement on a large scale in most LMICs due to the high cost of infrastructure and inadequate numbers of qualified cytotechnologists and pathologists to read cytologic smears. The etiologic link between persistent high-risk human papillomavirus (hr-HPV) infection and cervical cancer, together with knowledge about the natural history of these oncologic HPV types and their epidemiology has revolutionised cervical cancer screening, with most countries moving toward primary screening with HPV DNA testing.

Typically, screening with HPV DNA testing is sequential, with triaging or follow-up of hr-HPV-positives with visual inspection methods, such as colposcopy or visual inspection with acetic acid (VIA), in the absence of colposcopy. Because often HPV test results are not immediately available in screening units as a point-of-care test, this sequential approach to screening requires that women visit screening centres at another (sometimes multiple) times. This may be overcome by performing concurrent HPV DNA testing and visual inspection [4]. At follow-up of hr-HPV-positive women, the visual inspection method (colposcopy or VIA) aims to examine the transformation zone (TZ) where almost all cancers develop [5, 6]. This TZ moves into the endocervical canal as a woman ages (usually after 45 years); thus, the entire TZ is not visible for some women, meaning some (pre)cancerous lesions may be missed with colposcopy or VIA.

Women with TZ type 3 (where the entire TZ is not visible) pose a challenge to screening worldwide [7]. One way to overcome this challenge is to use a cytology/Pap smear cytobrush to sample the endocervical canal; this may pick up (pre)malignant cells from the TZ, which would otherwise not be visible or assessable on visual inspection. This may require yet another visit. ‘*Tritesting*’, where at the same visit, a sample is taken for HPV DNA testing and cytology (especially useful if the HPV DNA test is positive and the woman has TZ type 3), and a visual inspection method (colposcopy or VIA) performed after the sample is taken, offers an opportunity to prevent multiple visits and potentially reduce the cost of screening, especially if women have to travel long distances for screening.

Concurrent screening with HPV DNA testing, cytology and visual inspection (VIA or colposcopy) has been evaluated in various combinations in the context of research [8–12]. To the best of our knowledge, however, no prior study has evaluated the tritesting approach in routine clinical work, especially regarding its potential to reduce loss to follow-up in screening. The primary aim of this study was to present our experience with tritesting at the Cervical Cancer Prevention and Training Centre (CCPTC) in Catholic Hospital, Battor in the North Tongu District of the Volta Region of Ghana. Secondarily, we determined the prevalence of hr-HPV infection, abnormal cytology and cervical lesions among women screened using this approach.

## Materials and methods

### Study design

This descriptive retrospective cross-sectional study was conducted to determine the prevalence rates of hr-HPV infection, abnormal cytology and cervical lesions among women screened using ‘*tritesting*’, defined as the performance of HPV DNA testing, cytologic testing and visual inspection (VIA or mobile colposcopy) in a single session at the CCPTC between April 2019 to April 2023.

### Ethical considerations

The study complied with the Declaration of Helsinki (1964) and its later amendments. Verbal informed consent was sought from the women prior to questionnaire administration, sample collection and cervical screening. Ethical clearance was given by the Ethical Review Committee of the Catholic Hospital, Battor (approval no. CHB-ERC-002/07/19) to publish the findings of this study retrospectively.

### Study settings and participants

This study involved the screening of 236 women aged ≥21 years at the clinic (*n* = 209) and on outreaches (*n* = 27) by CCPTC staff between April 2019 to April 2023. The screening sessions were held at the CCPTC for women who visited the clinic and also on outreaches after health education exercises. Women who were willing and screened were included in the study.

The North Tongu District, which has its capital as Battor, is located along the Volta River and has a majority of its communities interspersed along the river. The district is mainly accessible by road and water (the Volta River via boat and canoe). Road networks are difficult to ply, especially during the rainy season. The district comprises about 310 communities, many of which are hard-to-reach areas. There are 29 health facilities in the district: 1 hospital (Catholic Hospital, Battor) which serves as the district hospital, 1 polyclinic, 6 health centres, 20 community health planning and service compounds and 1 private clinic. Catholic Hospital, Battor serves as the district hospital in the North Tongu District and serves a rural population but also sees many patients from nearby urban settings. The CCPTC was established in 2017 and offers cervical cancer prevention services and training to health workers (nurses, midwives, physician assistants and medical doctors) to offer cervical cancer prevention services in their respective areas of practice.

### Data collection

A structured questionnaire designed by the CCPTC and used routinely at the clinic and on outreaches was used to capture data of women screened. The data were collected by nurses after educating the women on the screening procedures and seeking verbal informed consent. All study data were captured and managed in REDCap version 11.0.3 (Vanderbilt University, Nashville, TN, USA) [13] and stored in secure databases hosted by the CCPTC. Prior to the analyses, the data were queried, extracted and converted into an Excel spreadsheet (Excel, Microsoft Corporation, Redmond, WA, USA) and manually cross-checked for accuracy. To ensure privacy and anonymity, all data were deidentified before the analyses.

### Study variables and outcomes

Sociodemographic data included age, educational status, marital status, parity, occupation, level of income, residence, National Health Insurance Scheme coverage, religion and parity. Data regarding self-reported risk factors were also collected, including details on current and past contraceptive use, smoking status (ever or current) and HIV status. The outcomes of interest were a positive hr-HPV DNA test, abnormal cervical cells on cytology (conventional or liquid-based), or the presence of cervical lesions on visual inspection (VIA or mobile colposcopy).

### Cervical HPV specimen collection, VIA and enhanced visual assessment (EVA) colposcopy

Ghana does not have a national cervical precancer screening programme so women pay out-of-pocket to get screened. While we generally start HPV DNA testing from the age of 25 years at our centre, we educate women about HPV and cervical cancer before screening. Women are made to understand that it takes 10–15 years, sometimes longer, from hr-HPV infection to cervical cancer. Some younger (age less than 25 years old) parous women (with early coitarche) who assess their risk sometimes opt for HPV DNA testing, and we do not deny them when they are ready to pay for it themselves.

All participants included in the study were screened using a combination of three cervical screening methods in the same setting and a single visit. The women opted to undergo this combination of tests due to convenience, affordability and turnaround time. Cervical screening was performed by trained nurses after positioning the woman in a dorsal lithotomy position and the cervix exposed using a sterile vaginal speculum.

Cervical samples for liquid-based cytology were taken using a Cervex-Brush® (Rovers Medical Devices BV, Oss, Netherlands) and fixed using PreservCyt (ThinPrep). Cervical samples for conventional cytology were obtained using an Ayre spatula and cytobrush, smeared on a glass slide, and immediately fixed with 92% alcohol. For women who underwent conventional cytology, samples for HPV DNA testing were taken using another cytobrush or cotton-tipped applicator which was then placed in a sample collection tube to be sent to the laboratory. For women who underwent liquid-based cytology, the liquid-based sample (ThinPrep) was split and part (4 mL) was submitted to the laboratory for testing. The choice of conventional or liquid-based cytology depended on the kits available at the time; however, liquid-based cytology was preferred. Catholic Hospital, Battor does not have an in-house cytotechnologist/pathologist; samples for cytology were sent out to a pathologist at another institution 100 km away. The results were available in a week.

In the same session, mobile colposcopy or VIA was performed by trained nurses after samples for cytology and HPV DNA testing had been taken. During the procedure, a mobile colposcope, the EVA system (MobileODT, Tel Aviv, Israel) which is built around a smartphone, and operated through an app was used to perform colposcopy, during which colposcopic images were captured before and after applying 5% acetic acid. VIA was performed for some women, during which the cervix was examined under a good source of light for abnormal changes after applying 5% acetic acid and waiting for 90–120 seconds. Findings on colposcopy were reported appropriately using the International Federation of Cervical Pathology and Colposcopy (IFCPC) terminology by recording whether the colposcopy was adequate, the type of TZ type seen and the presence of clinically significant cervical lesions [14]. The results of VIA were described as either ‘positive’ if aceto-white lesions were seen at the TZ or ‘negative’ otherwise. Women with cervical lesions on VIA or colposcopy were offered treatment by way of thermal coagulation or loop electrosurgical excision procedure (LEEP).

### Definitions of TZ types

TZ types observed at colposcopy were classified according to the IFCPC 2011 criteria [14], detailed as follows:

TZ type 1: The entire circumference of the squamocolumnar junction is visible; fully ectocervical.

TZ type 2: The entire circumference of the squamocolumnar junction is visible; partly or fully endocervical.

TZ type 3: The entire circumference of the squamocolumnar junction is not visible; partly or fully endocervical.

### Laboratory processing of cervical specimens and HPV DNA assays

Depending on the test platform available at the time, cervical samples obtained via the aforementioned procedures were processed and assayed using the GeneXpert HPV system (Cepheid, Sunnyvale, CA, USA), AmpFire HPV system (Atila BioSystems, Inc., Mountain View, USA), or MA-6000 HPV assay system (Sansure Biotech Inc., Hunan, China). All tests were performed at the central laboratory of the Catholic Hospital, Battor according to the manufacturer’s instructions as detailed elsewhere [15–19].

GeneXpert assays were performed using cervical specimens in PreservCyt or ThinPrep liquid cytology specimens whereas the AmpFire and MA-6000 assays were run on genital swabs (dry brushes, swabs, or PreservCyt/ThinPrep). Since the GeneXpert IV module system was used at the central laboratory of Catholic Hospital, Battor, GeneXpert tests were run singly or in batches of four, with a sample-to-answer time of a little under an hour. AmpFire and MA-6000 tests were similarly performed singly or in batches of up to 94 with positive and negative controls, with sample-to-answer times of approximately 2 hours each. In terms of genotypes detected and their categorisation, the GeneXpert assay specifically identifies HPV 16 and 18/45, and collectively identifies HPV 31, 33, 35, 39, 51, 52, 56, 58, 59, 66 and/or 68 as *other* hr-HPV type(s). The AmpFire and MA-6000 tests were performed in their semi-quantitative modules, which specifically identify HPV 16 and 18, and collectively identifies 31, 33, 35, 39, 45, 51, 52, 53, 56, 58, 59, 66 and/or 68 as *other* hr-HPV type(s). Although full-genotype modules are available, they are more expensive and have lower throughputs [20], and so were not generally used for the participants of this study.

### Strategy for treatment and follow-up

**Figure 1.**
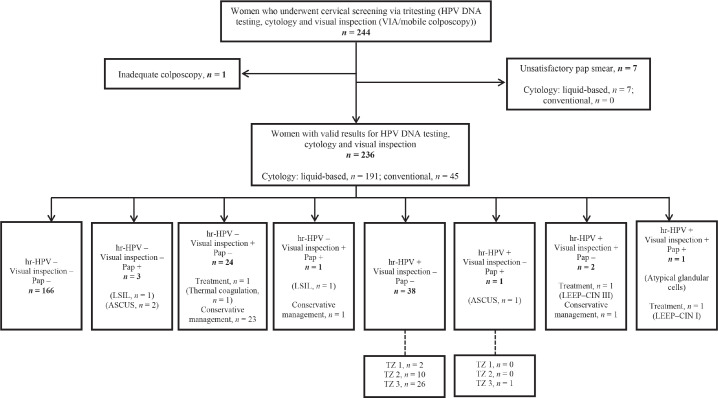
Flowchart for cervical precancer screening among women via ‘tritesting’ (hr-HPV DNA testing, visual inspection and cytology in a single visit). hr-HPV, high-risk human papillomavirus; LSIL, low-grade intraepithelial lesion; Pap, Papanicolaou test; TZ, transformation zone; LEEP, loop electrosurgical excision procedure; ASCUS, atypical squamous cells of undetermined significance; CIN, cervical squamous intraepithelial neoplasia.

The women were managed following the institutional algorithm for sequential screening with HPV DNA testing, colposcopy and cytology used at the CCPTC, which has been presented in a prior publication [21]. The standard approach was to wait for all results (HPV DNA and Pap test results took about 1–2 weeks) before treatment even if a lesion was observed during VIA or mobile colposcopy. Women with a Pap result of high-grade squamous intraepithelial lesion were recalled for LEEP even if no lesion was seen on the cervix, especially those with type 3 TZs [22]. Women with minor changes (thin acetowhitening) on visual inspection with a Pap result of low-grade squamous intraepithelial lesion, atypical squamous cells of undetermined significance (ASCUS) or negative for intraepithelial lesion or malignancy were asked to undergo follow-up in 1 year. There was the option, after discussion with women with cervical lesions (major/minor changes) on visual inspection, to have a ‘see and treat’ approach, where they undergo ablative treatment (thermal coagulation) even before the results of HPV DNA and Pap tests came in. Such women were required to satisfy the criteria for ablation (no suspicion of invasion and upper limit of the lesion seen) and made to understand that this may result in overtreatment as not all acetowhite lesions are cervical intraepithelial neoplasia (CIN) [4]. If they could afford the cost of histopathology and consented to it, a biopsy of the lesion would be taken and treatment deferred till the histopathology report was in (Figure 1).

### Assessing the setting-specific need for recall among women screened via concurrent testing

**Figure 2.**
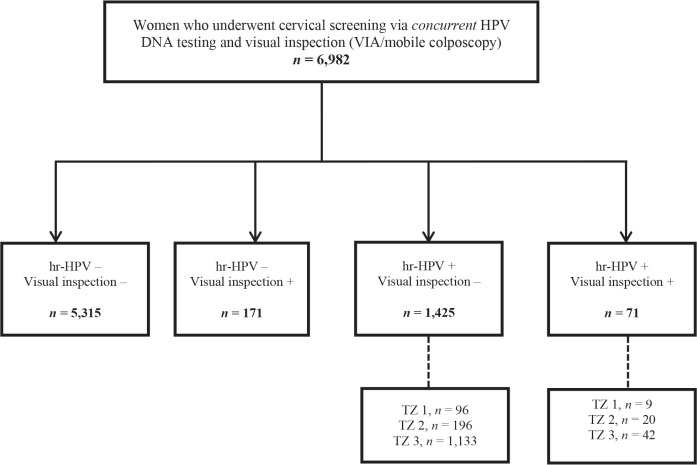
Flow chart for concurrent screening with hr-HPV DNA testing and visual inspection (EVA mobile colposcopy or VIA) during the study period. VIA, visual inspection with acetic acid; hr-HPV, high-risk human papillomavirus; TZ, transformation zone.

As concurrent testing (defined as *combined* HPV DNA testing and a visual inspection method (VIA or mobile colposcopy) in a single visit) is routinely performed at the CCPTC, we additionally evaluated the number of women screened via this approach (Figure 2) during the study period. The number needed to have been recalled was assessed among those who tested hr-HPV positive but visual inspection ‘negative’. Among these, we calculated the rate of loss to follow-up after recalling those with type 3 TZs to return for follow-up Pap tests.

### Statistical methods

The distributions of continuous and discrete data such as age and parity were evaluated using the Kolmogorov–Smirnov test. Nominal variables such as education level, marital status and the presence of hr-HPV infection are presented as counts and percentages. Symmetrically distributed continuous variables are presented as means with their standard deviations (SDs), whereas those with non-normal distributions are presented as medians with their interquartile ranges (IQRs). Positivity rates recorded on hr-HPV DNA testing, cytologic testing and visual inspection (VIA or mobile colposcopy) are presented in rate form, with 95% Clopper–Pearson confidence intervals (CIs). These positivity rates are further disaggregated by testing platform and type of cytologic test. Due to the low numbers of women subjected to GeneXpert HPV testing (*n* = 1) and screening with VIA (*n* = 3), we did not disaggregate hr-HPV positivity and visual inspection ‘positivity’ by these variables, respectively. Age and parity distributions of the women by outcomes of hr-HPV DNA testing, visual inspection and cytologic testing were compared using the Student *t*-test and the Wilcoxon rank-sum test, respectively, and are depicted in nested boxplots. We explored the association between hr-HPV infection and visual inspection ‘positivity’ and selected variables using univariable and multivariable binary logistic regression. Missing data were handled using multiple imputation by chained equations with ten iterations and the results were combined according to Rubin’s rules. For hr-HPV positivity, we used an arbitrary threshold of *p*-value = 0.25 for variable entry into the multivariable analysis and present odds ratios (ORs) and adjusted ORs (aORs) with their 95% CIs. For visual inspection ‘positivity’, we present ORs and age-aORs with 95% CIs. All statistical analyses were performed using Stata version 17.0 (StataCorp LLC, College Station, TX, USA). Hypothesis tests were two-tailed and performed at an alpha level of 5%.

## Results

### Recruitment and selection of study participants

Figure 1 shows a flow chart of participant selection, screening and outcomes. Two hundred and forty-four women consented to undergo tritesting during the study period. Among these, seven women had unsatisfactory Pap smears and one had inadequate colposcopy. Thus, the final dataset comprised 236 women with complete data for outcomes, including valid results for HPV DNA testing, visual inspection (VIA or EVA mobile colposcopy) and cytology (liquid-based cytology, *n* = 191; conventional Pap smear, *n* = 45).

### Sociodemographic and clinical characteristics of study participants

The participants had a mean age of 39.1 (SD, 10.9) years (range, 21–85) and a median parity of 1 (IQR, 0–2). Forty-seven per cent of the participants were married, 34% were single, 8% had a steady partner, 6% were divorced and 5% were widowed. A large majority (72%) had completed at least a secondary level of education and most (87%) earned an income, more than half (54%) of whom had a monthly income above 500 cedis (approximately USD 84.4, based on a median conversion rate of USD 1 = 5.92 Ghana cedis during the period under study). None of the participants reported ever smoking, 20% had used contraceptives in the past and 4% were using some form of contraceptive at the time of tritesting. A majority (55%) did not know their HIV statuses, 44% reported a negative HIV status and 1% were HIV-positive. In terms of medical history, the most commonly reported condition was hypertension (15%), followed by tuberculosis (8%), diabetes (3%), asthma (3%) and sickle cell disease (1%). Only one woman in the entire cohort had received prior HPV vaccination. Slightly more than half (*n* = 122, 52%) of the participants had undergone cervical screening in the past, three (2%) of whom were treated after screening (two by LEEP and one by thermal coagulation). At screening, 2% and 1% of women showed abnormal findings on gross vulval and cervical inspection, respectively, while none showed vaginal wall abnormalities.

### Participant triaging, outcomes of cervical screening and treatment of screen-positives

Table 1 also shows the prevalence rates of hr-HPV infection, cervical lesions on visual inspection and positive results on cytology (defined as a finding of ASCUS or worse). The overall hr-HPV positivity rate was 17.8% (95% CI, 13.1–23.3) and did not differ by the testing platform used (MA-6000, 16.0% versus AmpFire, 23.3%; *p*-value = 0.203). On visual inspection, clinically significant cervical lesions were seen in 11.9% (95% CI, 8.0–16.7) of the women, a value of which increased slightly to 12.0% (95% CI, 8.1–16.8) when considering only women screened using an EVA mobile colposcope. Overall, cytology yielded findings of ASCUS or worse in 2.5% (95% CI, 0.9–5.5) of the women, disaggregated as 3.1% (95% CI, 1.2–6.7) among 191 women who underwent liquid-based cytology, while none (0%) of the 45 women who underwent conventional Pap smears showed significant findings.

Among six women who showed positive findings on cytology, ASCUS was found in three cases, low-grade intraepithelial lesions were seen in two cases, and atypical glandular cells in one case. Following visual inspection, treatment was performed in three women (thermal coagulation in one woman and LEEP in two women). Histopathology following LEEP revealed CIN I in one woman (who tested positive on all screening methods) and CIN III in another woman (who tested hr-HPV-positive and visual inspection ‘positive’ but cytology-negative). The remaining 25 women who screened ‘positive’ on visual inspection were managed conservatively as they had minor changes (thin acetowhitening, which we believed could regress spontaneously) (Figure 1).

### Exploratory analysis of factors associated with positive findings on hr-HPV testing, visual inspection and cytology among women subjected to cervical screening via ‘tritesting’

**Supplementary Figure 1.**
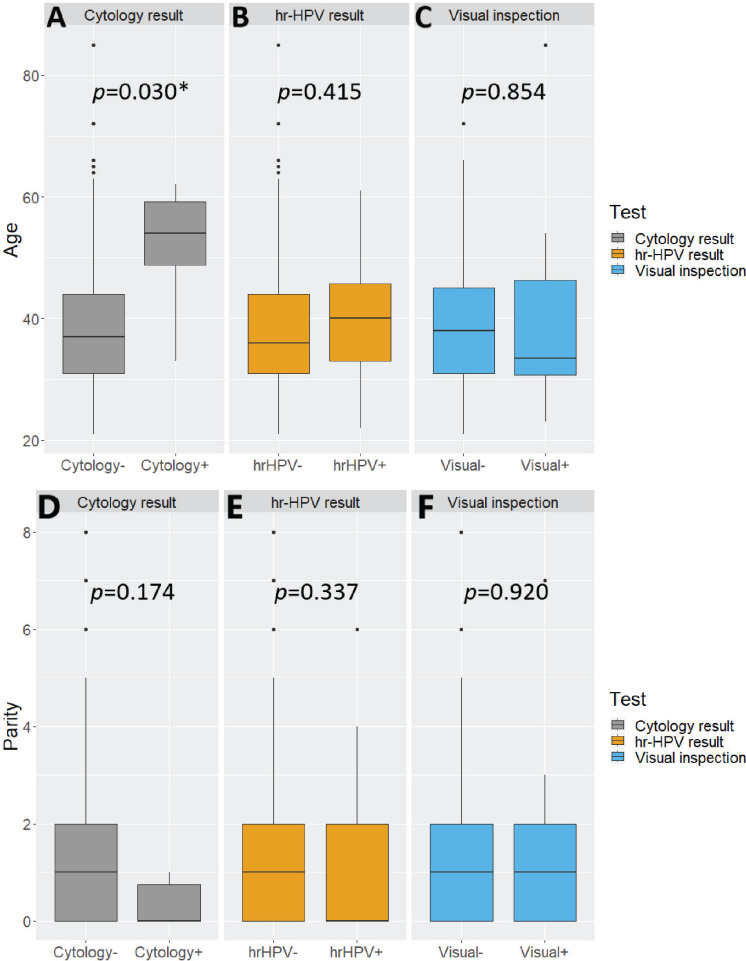
Nested boxplots showing the distributions of (A–C): age and (D–F): parity by test result among women who underwent cervical precancer screening via ‘*tritesting*’ (combined hr-HPV DNA testing, visual inspection and cytology). hr-HPV, high-risk human papillomavirus. ^*^Statistically significant.

hr-HPV-positive women tended to be slightly older than their hr-HPV-negative counterparts but without statistical significance between the two subgroups (Student *t*-test *p*-value = 0.415). A similar observation with a wider and statistically significant age gap was seen with cytology (Student *t*-test *p*-value = 0.030). The opposite was the case with visual inspection, with screen ‘positive’ women being younger than their visual inspection ‘negative’ counterparts, without statistical significance (Student *t*-test *p*-value = 0.854) (Supplementary Figure 1A–C). The distributions of parity were not significantly associated with positivity on cytology, hr-HPV testing, or visual inspection (Wilcoxon rank-sum test, *p*-values = 0.174, 0.337 and 0.920, respectively) (Supplementary Figure 1D–F).

In the logistic regression analysis, factors that were independently associated with an increased likelihood of hr-HPV positivity were age ≥ 39 years (aOR = 2.56; 95% CI, 1.19–5.52; *p*-value = 0.016), tertiary level of education (aOR = 2.13; 95% CI, 1.02–4.44; *p*-value = 0.044) and current contraceptive use (aOR = 4.66; 95% CI, 1.14–19.10; *p*-value = 0.033) (Table 2). Other variables entered into the multivariable analysis for hr-HPV positivity, including earning an income, past contraceptive use and prior cervical screening were not significantly associated with hr-HPV infection. None of the aforementioned factors also showed statistically significant associations with visual inspection ‘positivity’ in the univariate and age-adjusted logistic regression analyses (Table 2).

### Setting-specific loss to follow-up among women screened via concurrent testing during the study period

During the period under study, 6,982 women were screened using concurrent testing (combined HPV DNA testing and a visual inspection method (VIA/mobile colposcopy)) (Figure 2). Among these, 71 (1.0%) tested hr-HPV positive and visual inspection ‘positive’, while 1,425 (20.4%) tested hr-HPV positive but visual inspection ‘negative’. In the latter group of visual inspection ‘negative’ but hr-HPV screen positives, the commonest type of TZ was type 3, which was found in 1,133 out of 1,425 (79.5%; 95% CI, 77.3–81.6). After recalling these women to present for follow-up Pap tests, 267 returned, implying a loss-to-follow-up rate of 76.4% (95% CI, 73.9–78.9).

## Discussion

This paper primarily aims to share our experiences with tritesting (hr-HPV testing, VIA or mobile colposcopy and cytology in a single visit) in the context of routine clinical work done as an alternative to the ‘screen, triage and treat approach’ recommended by the World Health Organization (WHO), particularly as regards its potential to reduce loss to follow-up. We found an overall hr-HPV positivity rate of 17.8%, with no significant difference in detection rate between the testing platforms used. Approximately 12% of women subjected to tritesting showed clinically significant lesions on visual inspection. Cytologic findings of ASCUS or worse were seen in approximately 3% of the women. Histopathology following LEEP revealed CIN I (tritest positive) and CIN III (hr-HPV-positive, visual inspection ‘positive’, cytology-negative) in one woman each. Cytologic positivity was significantly associated with older age, whereas hr-HPV positivity and visual inspection ‘positivity’ were not. We also observed no significant associations between positive findings on hr-HPV testing, cytology or visual inspection and parity, level of education, earning an income and level of income.

The overall hr-HPV prevalence among our participants was much lower than the expected rate of 21.3% reported by the WHO for the general population of West African women [23, 24]. This rate was however very similar to the prevalence of 17.9% (95% CI, 16.7–19.0) our group found in a prior cohort of women subjected to concurrent cervical screening (hr-HPV testing and EVA mobile colposcopy in a single visit) using the same screening tools and test platforms [4]. The observed prevalence rate was again not too dissimilar to the aggregate positivity rate of 19.7% reported among community-dwelling Nigerian women when hr-HPV genotypes were classified in a similar manner (when recognised, probable and potential hr-HPV types were combined) as the qualitative versions of MA-6000 and AmpFire used in this study.

HPV DNA testing is generally more sensitive than cytology for detecting precancerous lesions. However, continued reports of hr-HPV-negative cytology-positive lesions have motivated the continued use of cotesting [25–27]. Cotesting (HPV DNA testing performed at the same time as cytology) has been recommended by several societies in the United States though there is a shift now toward primary screening with HPV DNA testing alone. Cotesting combines the advantages of the high sensitivity of HPV DNA testing and the high specificity of cytology. The main challenge with cotesting is increased cost. Also, screen positives have to be scheduled for follow-up with colposcopy which can lead to loss to follow-up. Concurrent HPV DNA testing with a visual inspection method (VIA or colposcopy) obviates the need for women to be recalled for follow-up when HPV DNA test results come in as positive [4].

Tritesting (combined HPV DNA testing, visual inspection (VIA or colposcopy) and cytology in a single visit) further reduces the loss to follow-up for screen positives (hr-HPV-positives or those who show cytologic findings of ASCUS or worse). Within the screening programme used at Catholic Hospital, Battor [4, 21] (most women undergo concurrent HPV DNA testing and a visual inspection method), women who test hr-HPV-positive with TZ type 3 (squamocolumnar junction not fully visible–partly or fully in the endocervical canal) are triaged to cytology to rule out a high-grade lesion in the endocervical canal [7]. Tritesting obviates the need for women to be recalled for another speculum examination to take another sample as the same sample (ThinPrep) can be used for both HPV DNA testing and cytology. Twenty-seven out of 39 hr-HPV-positive women (69.2%; 95% CI, 52.4–83.0) showed a type 3 TZ and would have needed to be recalled for a cytologic sample to be taken in a ‘see and triage’ approach with HPV DNA testing and a visual inspection method, thereby being potentially lost to follow-up.

The cost-effectiveness of tritesting depends on the individual and context of screening. While it generally increases the cost of screening for women, some women prefer this due to the advantage of a single visit to a health facility, and is more cost-effective, especially for women who travel long distances to access cervical screening services. Also, the cost of transportation and time required for follow-up with cytology is higher than the additional cost of cytology for some women. Again, while liquid-based cytology is generally more expensive than conventional cytology, we use low-cost cytology [28] which makes the cost similar to conventional cytology. With samples taken into a collection medium (ThinPrep), it is also possible to perform concurrent testing (HPV DNA testing and a visual inspection method (VIA or mobile colposcopy)). Women whose HPV DNA tests come out as hr-HPV-positive and who have TZ type 3 are then contacted. These women have the option of paying electronically for the liquid-based cytology to be performed using the previously collected sample without having to come to the clinic. This arrangement was put in place because women have to pay out-of-pocket for cervical precancer screening in Ghana. In other settings where women do not have to pay from their pockets for cervical precancer screening, ‘reflex cytology testing’ can be performed for only those who test hr-HPV-positive and have TZ type 3 on colposcopy or VIA, thereby reducing the cost of screening. The application of our tritesting approach was especially useful during the COVID-19 pandemic when it was necessary to limit visits to health facilities.

In terms of resource utilisation in our setting, apart from 3% to 5% acetic acid and cotton swabs, which are inexpensive, no additional materials are needed to perform visual inspection in the same setting as sample collection for HPV DNA testing and cytology. Thus, this approach does not significantly increase cost compared to an approach that requires a second visit when the HPV test or cytology returns positive findings, during which additional speculum, gloves, acetic acid and cotton swabs are needed. The additional time may be about 3–5 minutes per woman screened. An occasional challenge is bleeding from the ectocervix, especially for women with atrophy, which may make interpretation of visual inspection findings difficult. Screening with visual methods using artificial intelligence holds good promise in the near future [29–31]. This means that middle-cadre health workers, not only expert colposcopists can perform the visual inspection and get accurate results immediately. This will remove the bottleneck of a long training curve required to get adequately-trained colposcopists for the visual inspection aspect of tritesting.

In our approach at Battor, VIA and mobile colposcopy are performed by trained and experienced nurses [32]. While concurrent screening with HPV DNA testing, cytology and visual inspection (VIA or colposcopy) has been evaluated in the context of research [33], to the best of our knowledge, the present work is the first done in routine clinical work to evaluate this tritesting approach. Tritesting may not be cost-effective in a population-based/national cervical precancer screening programme in many LMICs, but in countries like Ghana where women have to pay from their pockets to get screened and may have to travel long distances for screening and follow-up, this may be cost-effective for some women and should be considered as an alternative to other methods. Where cost is a problem, HPV DNA testing using a liquid-based medium (instead of dry brushes), such that the same medium can be used for reflex cytology (only when the HPV test is positive and the woman has TZ type 3 on visual inspection) can be considered. Combining multiple tests can however strain healthcare facilities and lead to overcrowding, longer wait times and potential delays in accessing care for other health concerns, especially if a dedicated screening service is unavailable. In such a context also, facilities may struggle to allocate resources efficiently when overwhelmed with screening requests, which can affect the quality of care and patient experience.

As previously highlighted, two women in our study cohort were treated with LEEP. Both had major changes (dense acetowhitening) on colposcopy. One had a histologic diagnosis of CIN III while the other had CIN I (Figure 1). Twenty-six women had minor changes (thin acetowhitening) at colposcopy and were managed conservatively, to be followed up in 6 months or 1 year. Routine biopsies were not taken for the minor changes at colposcopy (thin acetowhite lesion) on discussion with the women, to reduce cost. Per our algorithms [4], some women still need to be recalled for a Pap smear if they test hr-HPV-positive and have TZ type 3, with the full circumference of the squamocolumnar junction not entirely visible. As shown in Figure 2, within the study period (between April 2019 to April 2023), 6,982 women were screened with concurrent HPV DNA testing and a visual inspection method, as routinely performed at the CCPTC [4]. Among these concurrently tested women, 1,133 out of 1,425 hr-HPV-positives had TZ type 3, which means they needed further evaluation of the endocervical canal with a Pap smear (or endocervical curettage). Despite efforts to contact these women for follow-up, we were only able to get 267 (23.6%) to return for Pap smears, implying a loss-to-follow-up rate of 76.4% (95% CI, 73.9–78.9). Tritesting would have reduced this high rate of loss to follow-up and the associated risk of potentially missing high-grade lesions or even cervical cancer in the endocervical canal.

Though it is possible to have the HPV test result on the same day (the GeneXpert platform, for example, runs the HPV test in 58 minutes without the need to batch samples), the HPV and the cytology test results are usually not available on the same day. Only the result of the visual inspection method (colposcopy or VIA) is guaranteed on the same day. Nevertheless, women may be offered treatment on the same day (‘see and treat’) after discussion with the screening team if there is a cervical lesion. This is especially important for women who experience difficulty getting to a health facility for follow-up. The limitation here is that the screening team cannot biopsy the lesion for histopathology to guide management. There may still be a benefit in using tritesting in these settings. For example, in the future management of these women, repeat HPV testing can give information on hr-HPV persistence or clearance. In future, point-of-care HPV/molecular testing and automated Pap smears which can be run at the point of care may make all three results available on the same day to guide decision making. In our work, 24 women (9.8%) screened positive on visual inspection but cytology-negative and hr-HPV-negative. These women could potentially have received treatment on the same day they were screened though they would not have been followed up if they had a primary screening with HPV DNA testing or cytology with only screen positives being triaged with a visual inspection method. Some of these women may be overtreated as the presence of acetowhitening, which is commonly the criterion for adjudging positivity on VIA or mobile colposcopy, may be due to immature squamous metaplasia, inflammation, subclinical papillomavirus infection or CIN. However, in a setting where women are unlikely to be screened many times in their lives, this approach is likely to prevent some cancers as there could be false negative HPV and cytology test results. It is important to note that this recommendation should be adapted to local contexts and resources. Decisions regarding the use of tritesting should take into account the specific circumstances and needs of a given region, as well as the woman in question. Local health authorities and policymakers should assess the alignment and potential misalignment of tritesting with WHO recommendations in the context of their healthcare systems and patient populations to make informed decisions about this cervical precancer screening strategy.

## Limitations

Our study had some important limitations. First, we used the qualitative versions of the MA-6000 and AmpFire HPV DNA tests, and so could not determine the exact hr-HPV genotypes present among hr-HPV-positive women. Also, because cervical screening is not covered by the National Health Insurance Scheme and women have to pay out-of-pocket to get screened, the average woman may not be able to afford tritesting. Again, although we did not assess the level of awareness of cervical cancer in the study cohort, more than half of the women had undergone previous cervical screening. This relatively high rate of prior cervical screening could indicate that this study cohort is much more aware of their risk of cervical cancer than most women in the general population, particularly as our previously studied cohorts have shown much lower rates of prior screening. In addition, our finding that 41.5% of women had completed tertiary education compared to the 10.4% rate recorded in the 2021 Population and Housing Census [34] indicates that our study cohort was a selected group. These characteristics limit the generalisability of our findings to the entire female population of Ghana.

## Conclusion

Although HPV DNA testing has higher sensitivity than cytology for detecting cervical precancers, continued reports of hr-HPV-negative cervical precancers and cancers motivate a combined approach in many settings. Here, we described our experience with tritesting as an alternative to other methods, particularly for women who prefer this due to the advantage of a single visit to a health facility and being more cost-effective if they have to travel long distances to access cervical screening services.

## Conflicts of interest

The authors declare that they have no conflict of interest.

## Funding

This work received no specific grant from any funding agency in the public, commercial, or not-for-profit sectors.

## Author contributions

Conceptualisation and study design: KE, ET and CMW; Screening and data collection: ET, CMW, ES, GBK, GT, FT, SK, SD, YTK and KE; Data management and formal analysis: NOME, SD, ET, ES, YTK, KAA, CMW and KE; Writing – original draft: NOME, ET, BHA, PKA and KE. All the authors read and approved the manuscript in its current form.

## Figures and Tables

**Table 1.** Sociodemographic and clinical characteristics of women who underwent cervical screening via ‘tritesting’ (hr-HPV DNA testing, visual inspection and cytology) (*n* = 236).

Sociodemographic variables	Estimate
Age, years; mean (SD)	39.1 (10.9)
Age group, *n* (%)	
<30	43 (18.2)
30–39	87 (36.9)
40–49	66 (28.0)
50–59	29 (12.3)
≥60	11 (4.7)
Parity, median (IQR)	1 (0, 2)
Religion, *n* (%)	
Christian	221 (93.6)
Muslim	11 (4.7)
Traditional	1 (0.4)
Missing	3 (1.3)
Marital status, *n* (%)	
Divorced	15 (6.4)
Married/cohabiting	110 (46.6)
Single	81 (34.3)
Widowed	11 (4.7)
Has a steady partner	18 (7.6)
Missing	1 (0.4)
Education level, *n* (%)	
No formal education	11 (4.7)
Elementary education	41 (17.4)
Secondary education	73 (30.9)
Tertiary education	98 (41.5)
Vocational/commercial/other	1 (0.4)
Missing	12 (5.1)
Monthly income level, GH¢; *n* (%)	
<100	13 (5.5)
100–250	54 (22.9)
250–500	16 (6.8)
>500	111 (47.0)
Prefer not to say	11 (4.7)
Does not earn an income	28 (11.9)
Missing	3 (1.3)
Past/current alcohol consumption, *n* (%)	26 (11.0)
Missing	21 (8.9)
Current/ever smoked, *n* (%)	0 (0.0)
Past/current contraceptive use, *n* (%)	51 (21.6)
**Clinical and screening characteristics**	
HIV status, *n* (%)	
Positive	3 (1.3)
Negative	103 (43.6)
Unknown	130 (55.1)
Past medical history	
Hypertension, *n* (%)	35 (14.8)
Diabetes mellitus, *n* (%)	8 (3.4)
Asthma, *n* (%)	7 (3.0)
Tuberculosis, *n* (%)	18 (7.6)
Sickle cell disease, *n* (%)	2 (0.9)
Previous HPV vaccination, *n* (%)	1 (0.4)
Missing	1 (0.4)
Previous cervical screening, *n* (%)	122 (51.7)
Previous cervical treatment, *n* (%)	3 (2.5)^b^
Vulval inspection findings, *n* (%)	
Normal	231 (97.9)
Abnormal	5 (2.1)
Vaginal inspection, *n* (%)	
Normal	236 (100.0)
Abnormal	0 (0.0)
Cervical inspection, *n* (%)	
Normal	234 (99.2)
Abnormal	2 (0.8)
Adequate for EVA mobile colposcopy^a^, *n* (%)	234 (100.0)
TZ type on EVA^a^	
1	22 (9.4)
2	56 (23.9)
3	151 (64.5)
Missing	5 (2.1)
hr-HPV-positive, % (95% CI)	17.8 (13.1–23.3)
MA-6000	16.0 (10.9–22.3)
AmpFire	23.3 (13.4–36.0)
Visual inspection ‘positive’, % (95% CI)	11.9 (8.0–16.7)
Mobile colposcopy	12.0 (8.1–16.8)
Cytology-positive, % (95% CI)	2.5 (0.9–5.5)
Liquid-based	3.1 (1.2–6.7)
Conventional^c^	0.0 (0.0–0.0)

SD, standard deviation; IQR, interquartile range; HIV, human immunodeficiency virus; hr-HPV, high-risk human papillomavirus; TZ, transformation zone

a Among 234 women who underwent EVA mobile colposcopy

b Among 122 women who had undergone prior cervical screening

c Among 45 women who underwent conventional Pap smears

**Table 2.** Exploratory logistic regression analyses of factors associated with hr-HPV positivity and visual inspection (EVA or VIA) ‘positivity’ among women who underwent cervical screening via ‘tritesting’.

Sociodemographic variables	hr-HPV positivity						Visual inspection ‘positivity’					
	Univariate models			Final multivariable model			Univariate models			Age-adjusted multivariable model		
	OR	95% CI	*p*-value	aOR	95% CI	*p*-value	OR	95% CI	*p*-value	aOR	95% CI	*p*-value
Age, years	1.01	0.98–1.04	0.453	-			1.00	0.96–1.03	0.832	-		
Age group, years												
<39 (Ref.)	1.00	-	-	1.00	-	-	1.00	-	-	-		
≥39	1.73	0.88–3.42	0.111	2.56	1.19–5.52	0.016^*^	0.78	0.35–1.73	0.540	-		
Religion												
Christian (Ref.)	1.00	-	-	-			1.00	-	-	1.00	-	-
Muslim/traditional	0.41	0.05–3.28	0.402	-			1.50	0.31–7.23	0.613	1.48	0.30–7.22	0.631
Marital status												
Divorced	1.45	0.42–5.01	0.552	-			-			-		
Married/cohabiting (Ref.)	1.00	-	-	-			-			-		
Single	0.63	0.29–1.38	0.249	-			-			-		
Widowed	0.89	0.18–4.41	0.885	-			-			-		
Has a steady partner	0.50	0.11–2.34	0.378	-			-			-		
Education level												
Tertiary	1.63	0.83–3.22	0.159	2.13	1.02–4.44	0.044^*^	1.03	0.46–2.32	0.938	1.02	0.44–2.40	0.955
Non-tertiary (Ref.)	1.00	-	-	1.00	-	-	1.00	-	-	1.00	-	-
Earns an income (yes/no)	0.48	0.19–1.18	0.110	-			1.16	0.33–4.12	0.821	1.13	0.31–4.13	0.849
Monthly income, GH¢												
≤500 (Ref.)	1.00	-	-	-			1.00	-	-	1.00	-	-
>500	1.70	0.75–3.84	0.200	-			1.28	0.53–3.10	0.577	1.20	0.46–3.05	0.718
Current alcohol consumption	0.99	0.27–3.63	0.993	-			0.47	0.06–3.69	0.471	0.46	0.06–3.70	0.472
Past contraceptive use	2.03	0.96–4.30	0.063	-			0.83	0.30–2.32	0.729	0.84	0.30–2.33	0.733
Current contraceptive use (yes/no)	3.30	0.89–12.25	0.075	4.66	1.14–19.10	0.033^*^	0.82	0.10–6.72	0.852	0.79	0.09–6.61	0.827
Past medical history												
Hypertension (Ref.)	1.00	-	-	-			1.00	-	-	1.00	-	-
DM/Asthma/TB/SCD	1.99	0.57–6.90	0.278	-			0.70	0.12–4.19	0.700	1.14	0.15–8.45	0.895
Previous cervical screening (yes/no)	0.65	0.33–1.27	0.208	-			0.93	0.42–2.04	0.848	0.93	0.42–2.04	0.849

hr-HPV, high-risk human papillomavirus; HIV, human immunodeficiency virus; CI, confidence interval; OR, odds ratio; aOR, adjusted odds ratio; DM, diabetes mellitus; SCD, sickle cell disease; TB, tuberculosis; Ref., reference category; EVA, enhanced visual assessment; VIA, visual inspection with acetic acid

* Statistically significant
